# ATP binding cassette and cholecystokinin A receptor genetic variations in gallstone susceptibility 

**Published:** 2017

**Authors:** Saman Milanizadeh, Seyed Reza Mohebbi, Mahsa Khanyaghma, Amir Houshang Mohammad Alizadeh

**Affiliations:** 1 *Gastroenterology and Liver Diseases Research Center, Research Institute for Gastroenterology and Liver Disease, Shahid Beheshti University of Medical Sciences, Tehran, Iran*; 2 *Basic and Molecular Epidemiology of Gastrointestinal Disorders Research Center, Research Institute for Gastroenterology and Liver Disease, Shahid Beheshti University of Medical Sciences, Tehran, Iran *

**Keywords:** gallstone, ATP binding cassette, cholecystokinin A receptor, polymorphism

## Abstract

**Aim:**

It was aimed to assess the association of four polymorphisms and relative haplotypes in the ATP binding cassettes and cholecystokinin A receptor (rs6720173, rs11887534, rs4148217, rs1800857) with the risk of gallstone.

**Background:**

Gallstone is a multifactorial disease. Besides high penetrance genes, low or moderate penetrance polymorphisms may increase susceptibility to gallstone.

**Methods:**

200 gallstone patients and 251 healthy controls were analyzed in a case-control association model. Genotyping was carried out by restriction fragment length polymorphism. Randomly 10% of samples underwent for direct sequencing to confirm results.

**Results:**

Heterozygote variant of rs11887534 demonstrated protective effect on the risk of gallstone susceptibility in males (P=0.013; OR=0.125; CI95%=0.048-0.325). In contrast, C/C genotype associated with gallstone susceptibility in females (P=0.004; OR=5.555 CI95%=1.975-10.632). Moreover, rs1800857 showed association only in females (P=0.019; OR=0.283; CI95%=0.099-0.811). Haplotype analysis for rs1800857 showed GC, CC and CA association with gallstone.

**Conclusion:**

The most imperative polymorphisms of contributing genes to gallstone were analyzed in this study and rs11887534 and rs1800857 appeared to be associated with gallstone, which is expected to be further veriﬁed in a larger cohort in the future.

## Introduction

 Gallstone prevalence has been found to be around 10-15% in adults in Europe and USA, about 6% in north India, and more than 64% in female American-Indians (1,2). Gallstone is a multifactorial disease that complex interactions of genetic and environmental factors are responsible for its predisposition (3). Several studies have shown that long standing gallstones can contribute in gallbladder carcinoma(4). Approximately 90% of gallstones in the gallbladder developed from cholesterol (5). One clinical reason for gallstone formation is excessive cholesterol secretion into the bile causing supersaturation of bile with cholesterol. A large family of transmembrane proteins is ATP binding cassettes (ABC) that participate in the energy dependent transport of wide variety of substrates including cholesterol. One important member of this family is ABCG8 that makes an obligate heterodimer with ABCG5 to form a functional transporter which expresses highly in canalicular membrane domain of hepatocytes, biliary ducts and gallbladder. Absorption and secretion of cholesterol to pump it into the bile is the main responsibility for this transporter (6,7). ABCG5 and ABCG8 genes, are close together on chromosome 2, are regulated with the same transcription factors and express in the same cells(8). Decrease in gallbladder motility is another important factor in gallstone formation (9). Cholecystokinin A (CCKA) is a peptide hormone which acts as a potent messenger for postprandial gallbladder contraction. Cholecystokinin A receptor (CCKA-R), the mediator of CCKA action, responds to non-sulfate cholecystokinin hormones. Impaired Bile secretion in patients with gallstone due to CCKA-R defects has been reported(10). Single nucleotide polymorphisms at some gene loci may influence the quality and/or quantity of gene product and can be a useful marker of individual risk factor for susceptibility to complex disease(11-13). Genome wide association study of gallstone patients and linkage study of affected sibs presented important polymorphisms in ABCG5/8 and CCKA-R genes that participate in susceptibility to gallstone disease. The most important polymorphisms among these are Q604E in ABCG5 (rs6720173) (14), D19H in ABCG8 (rs11887534) (15-16), T400K in ABCG8 (rs4148217) (17) and an intron1 polymorphism in CCKA-R (rs1800857) (9). 

We aimed to investigate the association of these four single nucleotide polymorphisms with susceptibility to gallstone disease and determine distribution of their genotypes among an Iranian population. 

## Methods

**Patients**

The population of this study consisted of 451 unrelated subjects. The present case control study comprised 200 symptomatic (Moderate to severe pain under the right side of the rib cage that may radiated through to the back or to the right shoulder, nausea, queasiness, vomiting and etc.) in patients with gallstone undergoing cholecystectomy who were attended the Research Center for Gastroenterology and Liver Disease (RCGLD), Shahid Beheshti University of Medical Sciences, Tehran, Iran (95 females and 105 males). The gallstone-free controls included 251 healthy volunteers (128 females and 123 males) recruited as a group without any family history of gallstone and no hepatic, gastrointestinal cholestasis that absence of gallstone proven by ultrasonography. Sampling performed from 2010 to 2012. All included subjects agreed with written informed consent before participating in this study and the protocol was approved by the Ethic Committee of Research Center for Gastroenterology and Liver Disease Shahid Beheshti University of Medical Sciences. Age below 18 or above 80 and not willing to participate were considered as exclusion criteria of the study. 5 ml of peripheral blood sample from subjects collected in ethylene diamine tetra acetic acid (EDTA) coated tubes and stored at -4ºC prior to DNA extraction.

**SNP selection criteria**

The analyzed polymorphisms were selected on the basis of data bases prepared on National Center for Biotechnology Information SNP Database (http://www.ncbi.nlm.nih.gov/projects/SNP/) and literature searches. Validated SNPs were selected with a minor allele frequency of greater than 1%, multiple citations in the literature and/or multiple independent submissions to the SNP databases.

**Genotyping**

DNA was obtained from whole blood using standard salting-out method described previously by Miller et al. (18). The first step for genotyping was amplification of the sequence containing polymorphic site using polymerase chain reaction (PCR). Primers for PCR designed through gene runner® software (http://www.generunner.net). PCR for amplification of each sequence was performed with 150 ng of genomic DNA, 0.2 mM dNTPs, 10pmol of each primer, 2.0 mM MgCl2, and 1 U of Taq polymerase, in a final volume of 25 μl. It was followed by restriction fragment length polymorphism (RFLP). Selection and designation of enzymes were carried out online by NEB cutter 2.0 software. Properties of primers and restriction enzymes are described in table I. PCR products were analyzed on 1.5% agarose gel electrophoresis and RFLP products were analyzed on 3% agarose gel electrophoresis. To visualize samples, gel staining was performed by ethidium bromide. To improve the genotyping quality and validation, 10% of samples were undergone direct sequencing and the results were reproducible, with no discrepancy.

**Table 1:** Primer sequences and enzymes

Primer	sequence	Tm	Length (after cut)	Restriction enzyme	recognition allele
rs6720173-F	5’- CTTTCACTACCTGCTAATGAGATG -3’	63	240 (150+90)	BpuEI	G
rs6720173-R	5’ – GAGATAAACCACACCTGACACTG – 3’				
rs11887534-F	5’ – TTATCTGATGTACCTTTAGCCAGCG – 3’	69	406 (274+132)	BfuI	C
rs11887534-R	5’ – ATGTCCTCACACTGCTTGATGTCC – 3’				
rs4148217-F	5’ – GTAAGGTGGCAGGCGACTC – 3’	64	245 (155+90)	Hpy99I	C
rs4148217-R	5’ – TAATAGAAAGGGCTTAATGTGATATAC – 3’				
rs1800857-F	5’-GTTGCCAGGACAGGAATCGTG -3’	70	221 (135+86)	PstI	T
rs1800857-R	5’-CGGTGATGACCAGCGTGTTTC -3’				

**Table 2:** Characterization of patients and controls

variable	Patients n (%)	Control n (%)
*total*	200	251
*sex*	105(55) 95(45)	123(48.3) 128(51.7)
male female		
*tobacco*	85(35) 115(65)	92(27.8) 159(72.2)
smoker non-smoker		
*mean age ±SD*	57.2±18.5	43.9±16.6

**Table 3:** Allelic frequency of genotypes

SNP ID	Model	Genotype	Total number	Case n(%)	Control n(%)	OR (95% CI)	p-value
Rs11887534	Codominant	G/G	229	70(22.2)	159(77.8)	ref.	ref.
		G/C	141	83(66.7)	58(33.3)	0.173(0.089-0.336)	0.998
		C/C	81	81(100)	0(0)	0.678(0.135-1.265)	0.018
	Dominant	G/G	291	84(31.7)	181(68.3)	ref.	ref.
		G/C+C/C	160	150(80.6)	36(19.4)	9.343(5.086-17.161)	0.002
	Recessive	G/G/+G/C	426	201(48)	217(52)	ref.	ref.
		C/C	25	33(100)	0(0)	0.598(0.054-2.145)	0.008
	Overdominant	G/G+C/C	316	117(39.3)	181(60.7)	ref.	ref.
		G/C	135	117(76.5)	36(23.5)	5.080(2.815-9.166)	0.001
	Alleles	G	599	222(30.6)	327(69.4)	ref.	ref.
		C	303	178(75.7)	125(24.3	0.215(0.092-0.415	0.023
Rs4148217	Codominant	C/C	222	91(37.4)	131(62.6)	ref.	ref.
		C/A	158	74(44.6)	84(55.4)	0.919(0.478-1.769)	0.919
		A/A	71	34(25)	37(75)	3.372(0.113-6.596)	3.372
	Dominant	C/C	279	136(49.5)	139(50.5)	ref.	ref.
		C/A+A/A	172	98(55.7)	78(44.3)	0.983(0.537-1.8.2)	0.957
	Recessive	C/C+C/A	444	232(52.1)	213(47.9)	ref.	ref.
		A/A	7	2(33.3)	4(66.7)	0.558(0.057-5.474)	0.558
	Overdominant	C/C+A/A	286	138(49)	144 (51)	ref.	ref.
		C/A	165	96(56.8)	73(43.2)	1.111(0.631-1.956)	0.716
	Alleles	C	652	282(39.1)	370(60.9)	ref.	ref.
		A	350	168(43)	182(57)	0.899(0.458-1.659)	0.809
Rs6720173	Codominant	G/G	240	113(44.3)	127(55.7)	ref.	ref.
		G/C	218	106(38.1)	132(61.9)	1.685(0.852-3.334)	0.134
		C/C	173	79(32.5)	94(67.5)	2.087(0.788-5.526)	0.139
	Dominant	G/G	190	110(56.4)	85(43.6)	ref.	ref.
		G/C+C/C	261	124(48.4)	132(51.6)	0.697(0386-1.257)	0.230
	Recessive	G/G+G/C	379	204(53.4)	178(46.6)	ref.	ref.
		C/C	72	30(43.5)	39(56.5)	0.656(0.307-1.405)	0.278
	Overdominant	G/G+C/C	262	140(53)	124(47)	ref.	ref.
		G/C	189	94(50.3)	93(49.7)	0.874(0.502-1.521)	0.633
	Alleles	G	517	234(42.3	283(57.7)	ref.	ref.
		C	385	166(35.7)	219(64.3)	1.578(0.723-3.452)	0.148
Rs1800857	Codominant	T/T	123	91(36.9)	132(63.1)	ref.	ref.
		T/C	146	68(43)	78(57)	0.546(0.274-1.087)	0.546
		C/C	81	41(53.3)	40(46.7)	0.571(0.146-2.242)	0.571
	Dominant	T/T	309	136(48.9)	142(51.1)	ref.	ref.
		T/C+C/C	142	98(56.6)	75(43.4)	1.678(0.915-3.078)	0.094
	Recessive	T/T+T/C	424	215(50.9)	207(49.1)	ref.	ref.
		C/C	27	19(65.6)	10(34.4)	1.580(0.510-4.893)	0.428
	Overdominant	T/T+C/C	282	154(50.3)	152(49.7)	ref.	ref.
		T/C	169	80(55.2)	65(44.8)	1.376(0.766-2.472)	0.286
	Alleles	T	593	250(38.2)	343(61.8)	ref.	ref.
		C	309	150(45.9)	159(54.1)	0.567(0.186-1.752)	0.245

Data adjusted for confounder elements such as age and gender.

**Figure 1:**
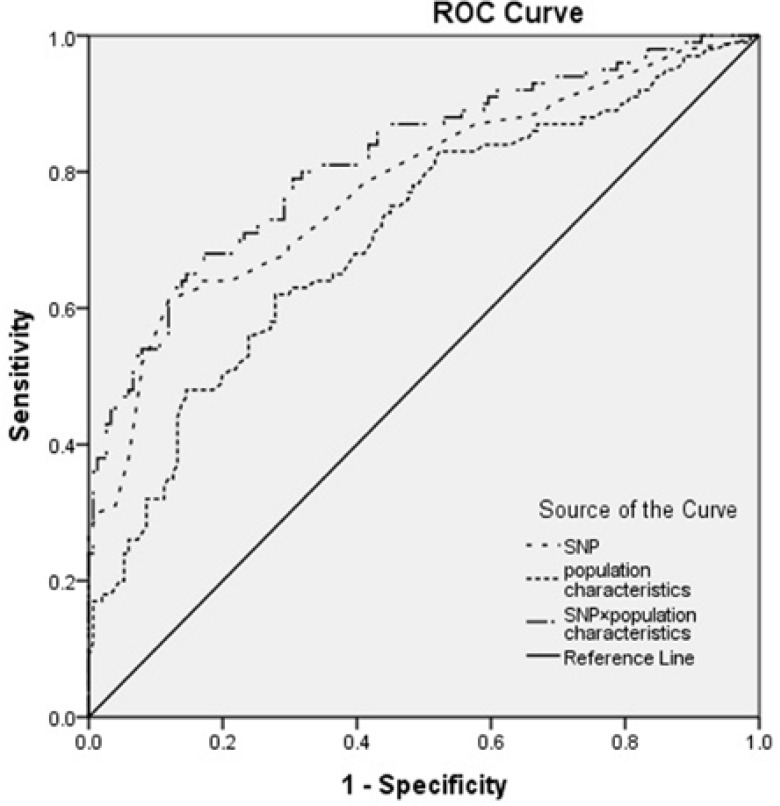
ROC curves for SNP risk, clinical features risk and clinical features × SNP risk. Diagonal segments are produced by ties.

**Table 4:** Stratified data for four SNPs in different sex groups

	Male			Female		
Genotypes	p-value	OR	CI 95%	p-value	OR	CI 95%
rs11887534						
G/G G/C C/C	ref. 0.013 0.249	0.125 1.453	0.048-0.325 0.731-2.952	ref. 0.532 0.004	2.437 5.555	0.527-4.142 1.974-10.632
rs4148217						
C/C C/A A/A	ref. 0.547 0.975	0.749 10.88	0.293-1.915 4.275-15-954	ref. 0.995 0.754	0.997 1.575	0.381-2.605 0.092-26.877
rs6720173						
G/G G/C C/C	ref. 0.171 0.119	2.045 2.864	0.734-5.697 0.763-10.752	ref. 0.36 0.505	1.574 1.732	0.596-4.157 0.345-8.698
rs1800857						
T/T T/C C/C	ref. 0.724 0.643	1.204 0.638	0.430-3.373 0.096-4.261	ref. 0.019 0.482	0.283 0.468	0.099-0.811 0.056-3.884

**Statistical analysis**

For categorical measures percentage and frequency were used, otherwise, in continuous measures meanSD were presented for descriptive statistic. For clarifying any deviation of cases and control groups from Hardy-Weinberg equilibrium ^2^ test was used. To assay statistical significant of age, student’s t test was used. For comparison of alleles and genotypes distribution chi-square test was performed. Relation of SNPs with gallstone formation was determined by binary logistic regression to calculate odds ratio (OD) and 95% confidence interval (CI) and data was adjusted for confounder factors such as age and gender. Statistical differences were considered as significant when p-values were less than 0.05. Sample size was adjusted to have >80% power to detect relative risk of SNP to gallstone susceptibility using GCTA-GREML Power Calculator(19). Logistic regression on the general mode was used to construct receiver-operating characteristic (ROC) curves and calculate the areas under the curve (AUCs) for more clarification of SNPs and disease association. We assessed classification performance of SNP risk, population characterization risk and SNP × population characterization risk using the ROC curve. Haplotype frequencies and linkage disequilibrium (D' and r^2^) were estimated based on the observed ABCG8 genotypes using CubeX software(18). Frequency threshold for rare haplotypes was set to 0.01. 

The putative functional effects of SNPs were determined Insilco by using online prediction tool F-SNP (http://compbio.cs.queensu.ca/F-SNP/ )(21). I-Mutant v2.0 webserver was used to predict protein stability upon mutation(22). Moreover, interaction network of all associated genes was determined by GENEMANIA (www.genemania.org). 

## Results

**Basic characteristics**

The baseline characteristics of gallstone patients and healthy controls are shown in table II. Within the population recruited in this study, 200 gallstone cases had higher mean age than 251 healthy controls. There were differences in sex distribution between case and control groups as patients were more male and healthy controls were more female. Chi-square test revealed association between disease and sex (p= 0.0136). Moreover, there were 49% women and 51% men totally. Smoking behavior did not demonstrate any effect on gallstone susceptibility (P= 0.264) while there were 30.7% tobacco smoker and 69.3% nonsmoker totally.

**Association of genotypes with gallstone**

Results for genotype distribution in co-dominant, dominant, recessive and over dominant models and allelic frequencies of four SNPs in the entire case-control groups are illustrated in table III. The genotype distribution of all SNPs showed no deviation from Hardy-Weinberg equilibrium. Analysis among rs6720173, rs11887534, rs4148217 and rs1800857 revealed the most frequent genotype for each SNP as G/G (42.2%), G/G (64.5%), C/C (61.8%) and T/T (62.5%) respectively. There are differences in allelic frequency between cases and controls of rs11887534 and rs6720173; however, other SNPs did not demonstrate any significant differences.

Logistic regression analysis was indicated association between rs11887534 heterozygote genotype and gallstone susceptibility (P=0.023; OR=0.173; CI95%=0.089-0.336). All data was adjusted for confounder elements such as age and gender. Data from stratified analysis of each SNP revealed that heterozygote genotype (G/C) of rs11887534 has a protective effect on gallstone susceptibility in males (P=0.013; OR=0.125; CI95%= 0.731-2.952). In contrast C/C genotype in females, increase gallstone susceptibility (P=0.004; OR=5.555; CI95%=1.974-10.632). So on, in female heterozygote genotype (T/C) of rs1800857 was demonstrated decrease in gallstone susceptibility (P=0.019; OR=0.283; CI95%=0.099-0.811). Stratified data for sexes is depicted on table 4.

**Haplotype analysis**

Haplotype analysis of ABCG8 revealed two protective haplotypes (CC and CA) and one that was associated with an increase in disease risk. The linkage disequilibrium (LD) was calculated on the basis of the genotype frequencies in both case and control populations. It was demonstrated that there was no linkage disequilibrium between the polymorphisms, as described in Table 5.

**ROC curve analysis**

To assess the ability of population study characteristics and SNP risk score to better classify gallstone susceptibility, ROC curve analysis was used (figure 1). We observed an AUC of 0.781 (95% CI= 0.721-0.842) for the SNP risk alone, compared with AUC of 0.708 (95% CI = 0.641-0.774) for population characteristics (sex, age and tobacco) risk and AUC of 0.819 (95% CI = 0.764-0.873) for the combination of population characteristics × SNP risk.

**In silico analysis**

Different SNP analysis revealed that rs6720173 can be the major contributing factor to gallstone. It changes secondary structure of protein and the result of this nucleotide exchange is deleterious. rs11887534 and rs4148217 have deleterious effect too. Rs1800857 demonstrates transcription factor binding site change. Table VI is showing in silico analysis of associated variants.

**Figure 2:**
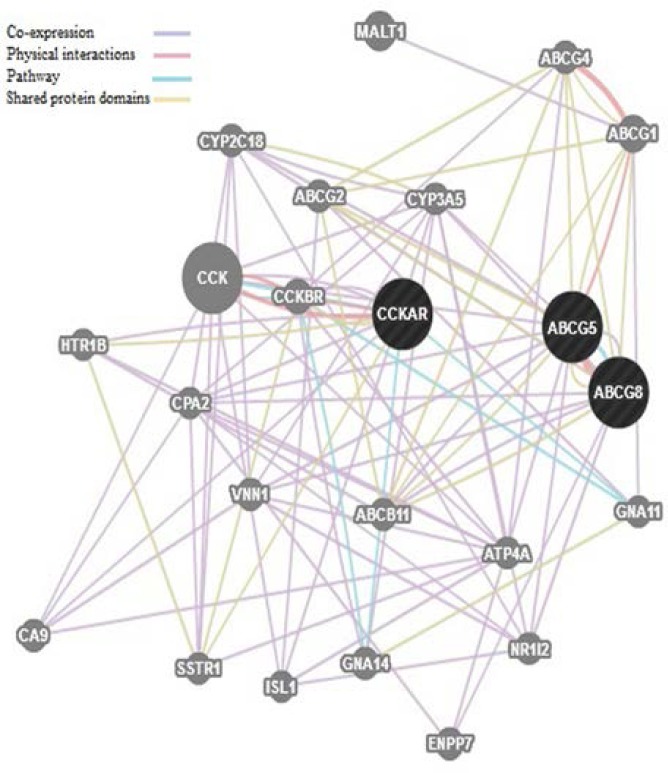
Interaction network of all associated genes. (Associated genes in bold

In silico analysis using I-Mutant v2.0 webserver showed decrease in protein stability due to SNP allelic changes of ABCG8 D19H, T400K and ABCG5 Q604E. To clarify communication of analyzed proteins, interaction network of ABCG8, ABCG5 and CCKAR proteins is depicted in figure.2 as co-expressions, physical interaction, pathway and shared protein domains.

**Table 5:** Haplotype frequencies and linkage disequilibrium statistics for ABCG8 polymorphisms

			Case			Control		
Haplotype	OR(05%CI)	p-value	frequency	D’	r^2^	frequency	D’	r^2^
GC	8.706(2.450-30.937)	0.001	0.5004	0.164	0.0116	0.7534	0.079	0.0024
GA	0.948(0.561-1.602)	0.842	0.1096			0.1666		
CC	0.117(0.065-0.210)	0.002	0.2846			0.0599		
CA	0.183(0.087-0.387)	0.004	0.1054			0.0201		

The degree of linkage disequilibrium between the two variants is shown as D’ for each group. p-value 0.05 was taken as statistically signiﬁcant for all the calculations

**Table 6:** Bioinformatics analysis

Genetic variant	Functional category	Prediction tool	prediction result	FS score
ABCG8 rs11887534	Protein coding	PolyPhen	Possibly damaging	0.339
		SNPeffect	Deleterious	
		Ensembl-NS	Nonsynonymous	
	Splicing-regulation	ESEfinder	Changed	
		ESRsearch	Changed	
	Transcriptional regulation	GoldenPath	exist	
ABCG8 rs4148217	Protein coding	PolyPhen	Possible damaging	0.424
		SNPeffect	Deleterious	
		Ensembl-NS	Nonsynonymous	
	Splicing regulation	ESEfinder	changed	
	Transcriptional regulation	GoldenPath	Exist	
	Post translation	OGPET	exist	
ABCG5 rs6720173	Protein coding	SNPeffect	Deleterious	0.533
		Ensembl-NS	nonsynonymous	
	Splicing regulation	ESRsearch	Changed	
		PESX	Changed	
		RESCUE ESE	changed	
CCKAR rs1800857	Transcriptional regulation	TFSearch	Changed	0.242
		GoldenPath	exist	

Ineffective results of SNP change in other databases are omitted

## Discussion

Within four SNPs we analyzed in this study only D19H variant showed significant correlation with gallstone formation, furthermore, our study revealed that CCKA-R intronic polymorphism was associated with disease only in females. Gallstone is one of the most prevalent disease by rate of 10%-20% in developed countries (15). The overall prevalence in Asia is lower, ranging from 3 to 15 per cent (23). This difference may relate to ethnicities and genetic factors that has been discussed previously.

In previous studies in Asia, susceptibility to gallstone differed between ethnicities and prevalence was higher in males (24). Many genetic details may interfere with disease susceptibility (25). Intestinal absorption of cholesterol and biliary excretion may be related to the ABCG5 and ABCG8 polymorphisms(6). In several studies D19H variant have been identified as the most prominent susceptibility factor for gallstone disease (14). In a research that conducted by Buch et al.(15) D19H variant, after adjusting for confounding risk factors such as sex, and age, was associated with gallstones in Germans and Chileans (OR=2.2; CI95%= 1.8–2.6). Srivastava et al.(26) reported that in a north Indian population, heterozygote genotype, and H allele increase susceptibility to gallstone disease. The variant H allele of the D19H polymorphism seemed to alter the expression or function of the ABCG8 transporter, ensuing increase of biliary cholesterol secretion and the accretion of gallstone (27). In the same study it was shown that the risk was more dominant in female cases and has been suggested that these differences might be due to a change in protein charge. By conferring OR rate as 2–3 and 7 for heterozygous and homozygous carriers, respectively, the D19H variant attribute to gallstone risk by means of 8–11% of total gallstones(28). Thorough transcript mapping, mutation detection and association analysis in ethnically different populations by von Kampen et al. demonstrated ABCG8 D19H as a likely causative variant for gallstone susceptibility.

Wang et al.(17) showed that the T400K polymorphism not D19H in ABCG8 might be associated with gallstone disease in Chinese males (OR=2.31; CI95%= 1.12–4.76). It was suggested that K400 carriers may have increased ABCG8 activity (16), resulting in maintain of plasma cholesterol low and in contrast increasing biliary cholesterol. In another study it was shown that phospholipid level was lower in male K400 carriers (17). Moreover, studies of T400K variant of ABCG8 gene showed that T allele might increase the risk of gallstone disease in specific populations(29).

In a research within siblings with gallstone, conducted by Acalovschi et. al. (30) it was revealed that D19H and Q604E SNPs were significantly associated with plasma lipid tract. In that study carriers of 604Q and D19H variants had high risk of gall stone formation. Their finding also was confirmed by Katsika et. al. (31). 

In our study age was associated with gallstone disease in T-test analysis (p=0.028, CI95% = 10.14-16.72). The ageing process enhances hepatic secretion of cholesterol and decreases bile acid synthesis. The ensuing bile super saturation with cholesterol can increase the risk of gallstone formation with ageing (32). As a result, all the regression tests in our study were carried out by adjusting the age.

CCKA-R dysfunction associated with several human disorders such as obesity, hyperphagia, hyperglycemia, and gallstone disease (33). Satu et. al. (34) showed that gallstone formation due to CCKA-R defects favored in the middle years of the infected persons lifespan. Our preliminary data in a previous study demonstrated that Rs11887534 polymorphism is associated with gallstone susceptibility (35). Association of CCKA-R intronic polymorphism with gallstone had been confirmed by Sirvastava et. al. In that study C/C homozygote genotype and C allele conferred high risk for gallstone disease with OR= 2.25 and 1.5, respectively. In the same research it was revealed that gender stratification, for this polymorphism, conferred a marginally significant risk for gallstone in female patients (OR = 2.15)(9). 

ROC curve analysis was demonstrated that the association between the SNPs and gallstone susceptibility is higher than clinical features. However, for more accuracy both factors should take into account. The in silico analysis of SNPs support the influence of genetic variants on proteins and showed variable changes in transcriptional regulation, splicing regulation, post translation and protein coding (Table VI). Moreover, interactome analysis of all associated genes depicted co-expression, physical interaction and shared protein domains of ABCG5 and ABCG8 (36). 

Compared with other studies, our study also confirmed that CKA-R intronic variant was affecting females. In the same way D19H variant of ABCG8 was attributed in gallstone formation neither T400K of ABCG8 nor Q604E of ABCG5. The differences between our findings and other studies have been come out by ethnicity segregation. Moreover, we suggest these findings to be confirmed in a large cohort study.

## Conflict of interests

The authors declare that they have no conflict of interest.

## References

[1] Minakari M, Molaei M, Shalmani HM, Mohammad Alizadeh AH, Jazi AH, Naderi N (2009). Liver steatosis in patients with chronic hepatitis B infection: host and viral risk factors. Eur J Gastroenterol Hepatol.

[2] Alizadeh AH, Ranjbar M, Ansari S, MirArab A, Alavian SM, Mohammad K (2006). Seroprevalence of hepatitis B in Nahavand, Islamic Republic of Iran. East Mediterr Health J.

[3] Mohebbi SR, Amini-Bavil-Olyaee S, Zali N, Noorinayer B, Derakhshan F, Chiani M (2008). Molecular epidemiology of hepatitis B virus in Iran. Clin Microbiol Infect.

[4] Sahebekhtiari N, Nochi Z, Eslampour MA, Dabiri H, Bolfion M, Taherikalani M (2011). Characterization of Staphylococcus aureus strains isolated from raw milk of bovine subclinical mastitis in Tehran and Mashhad. Acta Microbiol Immunol Hung.

[5] Saraç S, Atamer A, Atamer Y, Can AS, Bilici A, Taçyildiz İ (2015). Leptin levels and lipoprotein profiles in patients with cholelithiasis. J Int Med Res.

[6] Wang DQ (2007). Regulation of intestinal cholesterol absorption. Annu Rev Physiol.

[7] O'Connell K, Brasel K (2014). Bile Metabolism and Lithogenesis. Surg Clin North Am.

[8] Gylling H, Hallikainen M, Pihlajamaki J, Agren J, Laakso M, Rajaratnam RA (2004). Polymorphisms in the ABCG5 and ABCG8 genes associate with cholesterol absorption and insulin sensitivity. J Lipid Res.

[9] Mortazavi S, Zali M, Raoufi M, Nadji M, Kowsarian P, Nowroozi A (2002). The Prevalence of Human Papillomavirus in Cervical Cancer in Iran. Asian Pac J Cancer Prev.

[10] Ravikanth VV, Rao GV, Govardhan B, Sasikala M, Subramanyam C, Vivekananda Murthy HV (2016). Polymorphisms in UGT1A1 Gene Predispose South Indians to Pigmentous Gallstones. J Clin Exp Hepatol.

[11] Nazemalhosseini-Mojarad E, Haghighi A, Taghipour N, Keshavarz A, Mohebi SR, Zali MR (2011). Subtype analysis of Cryptosporidium parvum and Cryptosporidium hominis isolates from humans and cattle in Iran. Vet Parasitol.

[12] Karkhane M, Mohebbi SY, Azimzadeh P, Saeedi Niasar M, Sarbazi MR, Sharifian A (2016). Lack of association between interleukin 28B gene polymorphisms (rs8099917G/T, rs12979860 C/T) and susceptibility to chronic hepatitis C virus infection, Tehran, Iran. Gastroenterol Hepatol Bed Bench.

[13] Azimzadeh P, Romani S, Mirtalebi H, Fatemi SR, Kazemian S, Khanyaghma M (2013). Association of co-stimulatory human B-lymphocyte antigen B7-2 (CD86) gene polymorphism with colorectal cancer risk. Gastroenterol Hepatol Bed Bench.

[14] Kuo KK, Shin SJ, Chen ZC, Yang YH, Yang JF, Hsiao PJ (2008). Significant association of ABCG5 604Q and ABCG8 D19H polymorphisms with gallstone disease. Br J Surg.

[15] Buch S, Schafmayer C, Volzke H, Becker C, Franke A, von Eller-Eberstein H (2007). A genome-wide association scan identifies the hepatic cholesterol transporter ABCG8 as a susceptibility factor for human gallstone disease. Nat Genet.

[16] Yoon JH, Kuver R, Choi HS (2010). ABCG8 D19H polymorphism: a basis for the genetic prediction of cholesterol gallstone disease. J Gastroenterol Hepatol.

[17] Wang Y, Jiang ZY, Fei J, Xin L, Cai Q, Jiang ZH (2007). ATP binding cassette G8 T400K polymorphism may affect the risk of gallstone disease among Chinese males. Clin Chim Acta.

[18] Rostami-Nejad M, Villanacci V, Hogg-Kollars S, Volta U, Manenti S, Reza-Zali M (2013). Endoscopic and histological pitfalls in the diagnosis of celiac disease: A multicentre study assessing the current practice. Rev Esp Enferm Dig.

[19] Visscher PM, Hemani G, Vinkhuyzen AA, Chen G-B, Lee SH, Wray NR (2014). Statistical power to detect genetic (co) variance of complex traits using SNP data in unrelated samples. PLoS Genet.

[20] Gaunt TR, Rodríguez S, Day IN (2007). Cubic exact solutions for the estimation of pairwise haplotype frequencies: implications for linkage disequilibrium analyses and a web tool'CubeX'. BMC Bioinformatics.

[21] Lee PH, Shatkay H (2008). F-SNP: computationally predicted functional SNPs for disease association studies. Nucleic Acids Res.

[22] Bava KA, Gromiha MM, Uedaira H, Kitajima K, Sarai A (2004). ProTherm, version 4.0: thermodynamic database for proteins and mutants. Nucleic Acids Res.

[23] Portincasa P, Moschetta A, Palasciano G (2006). Cholesterol gallstone disease. Lancet.

[24] Rai R, Tewari M, Kumar M, Singh TB, Shukla HS (2011). Expression profile of cholecystokinin type-A receptor in gallbladder cancer and gallstone disease. Hepatobiliary Pancreat Dis Int.

[25] Marschall HU, Einarsson C (2007). Gallstone disease. J Intern Med.

[26] Srivastava A, Srivastava K, Choudhuri G, Mittal B (2010). Role of ABCG8 D19H (rs11887534) variant in gallstone susceptibility in northern India. J Gastroenterol Hepatol.

[27] Knab LM, Boller AM, Mahvi DM (2014). Cholecystitis. Surg Clin North Am.

[28] Farkas N, Karthigan R, Lewis T, Read J, Farhat S, Zaidi A (2017). A single centre case series of gallstone sigmoid ileus management. Int J Surg Case Rep.

[29] Rudkowska I, Jones PJ (2008). Polymorphisms in ABCG5/G8 transporters linked to hypercholesterolemia and gallstone disease. Nutr Rev.

[30] Acalovschi M, Ciocan A, Mostean O, Tirziu S, Chiorean E, Keppeler H (2006). Are plasma lipid levels related to ABCG5/ABCG8 polymorphisms? A preliminary study in siblings with gallstones. Eur J Intern Med.

[31] Katsika D, Magnusson P, Krawczyk M, Grunhage F, Lichtenstein P, Einarsson C (2010). Gallstone disease in Swedish twins: risk is associated with ABCG8 D19H genotype. J Intern Med.

[32] Zhang J, Prizment AE, Dhakal IB, Anderson KE (2014). Cholecystectomy, gallstones, tonsillectomy, and pancreatic cancer risk: a population-based case-control study in minnesota. Br J Cancer.

[33] Ding X, Lu CY, Mei Y, Liu CA, Shi YJ (2005). Correlation between gene expression of CCK-A receptor and emptying dysfunction of the gallbladder in patients with gallstones and diabetes mellitus. Hepatobiliary Pancreat Dis Int.

[34] Sato N, Miyasaka K, Suzuki S, Kanai S, Ohta M, Kawanami T (2003). Lack of cholecystokinin-A receptor enhanced gallstone formation: a study in CCK-A receptor gene knockout mice. Dig Dis Sci.

[35] Milanizadeh S, Mohamadalizadeh A, Azimzadeh P, Romani S, Khanyaghma M, Mohebbi SR (2013). Association of CCKAR Gene Polymorphism with Gallstone Disease. Med J Tabriz Univ Med Sci.

[36] Baghaei K, Shokrzadeh L, Jafari F, Dabiri H, Yamaoka Y, Bolfion M (2009). Determination of Helicobacter pylori virulence by analysis of the cag pathogenicity island isolated from Iranian patients. Dig Liver Dis.

